# Complete genome sequence of *Ligilactobacillus animalis* strain NP51

**DOI:** 10.1128/mra.00034-26

**Published:** 2026-02-27

**Authors:** Katherine P. Feldmann, Jonathan E. Beever, Phillip R. Myer

**Affiliations:** 1 Department of Animal Science, University of Tennessee 842097, Knoxville, Tennessee, USA; Nanchang University, Nanchang, Jiangxi, China

**Keywords:** *Ligilactobacillus*, probiotics, beef cattle

## Abstract

We report the complete genome sequence of *Ligilactobacillus animalis* strain NP51, a probiotic strain used extensively in the beef cattle industry.

## ANNOUNCEMENT

The genus *Ligilactobacillus* was established following recent reclassification of the *Lactobacillus* genus (1). Accordingly, the probiotic strain previously designated *Lactobacillus acidophilus* NPC 747 and later *Lactobacillus acidophilus* NP51 is now classified as *Ligilactobacillus animalis* NP51 (LANP51). Originally isolated from cattle, LANP51 is utilized in the feedlot sector as a pre-harvest intervention strategy due to its antagonistic activity against foodborne pathogens, including *Escherichia coli* O157:H7 (2, 3). Although a draft genome assembly has been previously deposited in GenBank, a complete genome sequence has not been reported (4). Here, we present the first complete genome assembly of LANP51.

LANP51 was obtained from Chr. Hansen A/S (part of Novonesis) and stored at –80°C. Lyophilized cells were resuspended in phosphate-buffered saline (PBS) (Thermo Fisher Scientific, USA) and allowed to equilibrate at room temperature for 10 min. The suspension was inoculated into 25 mL de Man, Rogosa and Sharpe (MRS; Merck, Germany) broth and incubated at 37°C for 18–24 h under aerobic conditions. Cultures were streaked onto MRS agar to verify purity and incubated at 37°C. A single colony was inoculated in 50 mL MRS broth at 37°C for 24 h.

Cells were harvested, washed in PBS, and pelleted. Genomic DNA was extracted using the Blood and Cell Culture DNA Kit (Qiagen, Germany) following the bacterial genomic DNA protocol with modifications recommended by Oxford Nanopore Technologies (ONT) (5). Subsequently, SPRI bead (Beckman Coulter, USA) cleanup was performed to remove DNA fragments under 2 kb per ONT’s recommendations (6).

Genomic sequencing was performed using the MinION Mk1D with Flongle adapter (ONT, UK) following the manufacturer's instructions. Unsheared genomic DNA was used for library preparation with the Ligation Sequencing Kit v14 (ONT) and NEBNext Companion Module v2 for ONT Ligation Sequencing (New England Biolabs, USA). Libraries were loaded onto a Flongle flow cell R10.4.1 using the Flongle Sequencing Expansion Kit (ONT). Sequencing was conducted with MinKNOW software v25.03.9 (24 h, 180 mV), and basecalling was performed with Dorado v7.8.3 (7, 8). A minimum quality (*Q*) score threshold of 9 was applied for read filtering. Default Dorado parameters were used for adapter trimming and error correction.

Approximately 101.33k reads (362.11 Mb) were generated, and 87.38k reads (348.21 Mb) passed quality filtering with an N50 read length of 6.29 kb. *De novo* assembly was performed using EPI2ME software v1.4.2 and wf-bacterial-genomes nextflow workflow v1.4.2 with Flye v2.9.5-b1801 default parameters for circularization and with no reorientation (9, 10). Polishing was performed with Medaka 2.0.0 (9). Gene prediction and annotation were performed with the NCBI Prokaryotic Genome Annotation Pipeline v6.10 (11–13).

Taxonomic identity was confirmed by average nucleotide identity (ANI) analysis using FastANI v1.34, which demonstrated 99.14% ANI to the reference genome (GenBank accession number CP047141.1) (14). The final genome assembly is 1,881,978 bp long, with a guanine-cytosine content of 41.2% and 168× coverage. A total of 1,925 genes were annotated, including 1,777 protein-coding sequences, 18 rRNA genes, 64 tRNA genes, 3 ncRNA genes, 62 pseudogenes, and 1 CRISPR array.

## Data Availability

The complete genome sequence has been deposited in GenBank under accession number CP195054.1. The raw sequencing reads have been deposited in the NCBI Sequence Read Archive under accession number SRR36833285. All data are available under BioProject accession number PRJNA1274020.
